# Same view, different lens: How intersectional identities reduce Americans’ stereotypes of threat regarding Arab and Black men

**DOI:** 10.1177/13684302231153802

**Published:** 2023-02-22

**Authors:** Veronica N. Z. Bergstrom, Jonathan Cadieux, Drishti Thakkar, Alison L. Chasteen

**Affiliations:** 1 University of Toronto, Canada; 2 Cégep Marie-Victorin, Canada

**Keywords:** Arab men, Black men, intersectionality, stereotypes

## Abstract

Because Black and Arab men may be stereotyped as hostile in different ways (i.e., physical vs. ideological), this study assessed whether an old age identity versus gay identity would reduce stereotypes related to hostility for Black and Arab men differently. We assessed whether the addition of an old age identity reduces hostile stereotype content more for Black men than for Arab men. In line with our hypothesis, an old age identity resulted in participants reporting fewer hostile stereotypes for Black men, but not for Arab men. We also assessed whether a gay identity reduces hostile stereotype content in the same way for Black and Arab men. As expected, a gay identity resulted in participants reporting fewer hostile stereotypes for both male groups. The present study demonstrates the importance of considering intersecting identities in person perception and highlights the unique challenges faced by men belonging to these intersecting groups.

## Introduction

As in many other countries, the demographic make-up of the United States is changing. Marking a historical moment in America, forecasts predict that by 2034 older adults will outnumber minors and this difference is expected to reach 15 million by 2060 (Vespa et al., 2020). These forecasts also predict that the older adult population will become increasingly ethnically diverse due to the growing population of people of color in the country as well as increased longevity (Vespa et al., 2020). Further, LGBTQIA+ identification is rising, particularly in younger age groups, with 15.9% of Generation Z reporting queer identification, compared with 9.1% of Millennials and 3.8% of Generation X (Jones, 2021). Given the growth of underrepresented groups in America, it is critical to understand how age and sexual orientation intersect with different racial/ethnic identities to influence reactions to members of these groups. Thus, in the present study, we investigate whether intersections between race/ethnicity and age as well as race/ethnicity and sexual orientation are characterized by different mental representations of men who belong to those intersecting groups.

In this work, we focus on two male groups that face negative stereotyping and continued discrimination in the United States: Black men and Arab men^1,2^ (e.g., Horowitz et al., 2019; Widner & Chicoine, 2011). In America, both Black and Arab men are stereotyped as hostile, but in different ways. Depictions of Black men as hostile often include associations with physical threats that require a degree of athleticism, such as robberies and assault (e.g., Smiley & Fakunle, 2016), whereas depictions of Arab men as hostile often include associations with terrorism and ideological threats^3^ (e.g., Saleem & Anderson, 2013). While it is possible that there are some instances in which Black and Arab men could be viewed as posing the alternative form of threat, the literature suggests that the most common types of hostility associated with each are physical hostility associations for Black men and ideological hostility associations for Arab men (Gordon et al., 1988; Guterman, 2013; Quillian & Pager, 2001; Saleem & Anderson, 2013; Shaheen, 2003; Smiley & Fakunle, 2016).

Given that hostility stereotypes about Black and Arab men differ, then it is also likely that intersections with other identities may lead to variability in the persistence of hostility stereotypes. For instance, previous research from Canada has demonstrated that an old age identity reduces hostile stereotypes associated with Black men (e.g., Kang & Chasteen, 2009), but it remains unknown whether an old age identity reduces hostile stereotypes for other racial or ethnic groups. Because Black and Arab men may be stereotyped as hostile in different ways (i.e., physical vs. ideological), it is possible that an old age identity may shift stereotype content in diverging ways for the two groups. For example, frail stereotypes associated with older adults (Hummert et al., 1994) may attenuate perceptions of physical hostility (as posed by Black men) but may not alter perceptions of ideological hostility (as posed by Arab men).

In contrast, a gay identity may reduce hostile stereotypes for both male groups. For example, non-gender-conforming stereotypes associated with gay men, such as being sensitive and high in communion (Petsko & Bodenhausen, 2019; Steffens et al., 2019), may attenuate perceptions of physical hostility (as posed by Black people); whereas a lack of religious extremism stereotypes associated with gay men may attenuate perceptions of ideological hostility (as posed by Arab people). Some prior work suggests that a gay identity may reduce hostile stereotypes for Black men (Petsko & Bodenhausen, 2019; Remedios et al., 2011); however, to our knowledge, whether a gay identity may reduce hostile perceptions for other racial or ethnic groups has not been explored. If hostile stereotypes about Arab men are driven by perceptions of ideological threat caused by religious extremism, then it is possible that a gay identity may attenuate hostile stereotypes of Arab men more than an old age identity.

To our knowledge, previous research has not investigated how an old age identity shifts stereotype content away from the superordinate categories of Arab men relative to Black men, nor whether stereotype content shifts in similar or diverging ways when sexual orientation is considered. Therefore, this study aimed to understand how stereotypes of Black and Arab men change when race/ethnicity and age as well as race/ethnicity and sexual orientation are simultaneously taken into account. In the following sections, we present evidence for why an old age stereotype may offset the hostile stereotype for Black men rather than Arab men, whereas a gay stereotype may offset the hostile stereotype for both racial/ethnic groups.

## Black Stereotypes

Stereotypes against Black people in the United States were established during the institution of slavery and still persist today (Feagin & Feagin, 1999; Plous & Williams, 1995; Taylor et al., 2019). Compared with arrest rates, United States law enforcement agencies overrepresent Black suspects in their Facebook announcements (Grunwald et al., 2022). This repeated pairing of Black people with criminality leads to a host of negative consequences. For example, exposure to network news is positively correlated with perceptions that Black people are poor and intimidating as well as positively correlated with racist attitudes (Dixon, 2008). In particular, Black men are considered the prototypical gender for their group and are more strongly associated with danger than Black women are (Thiem et al., 2019).

Black males are often negatively stereotyped as aggressive, angry, disobedient, hot-tempered, rebellious, and unintelligent (Devine, 1989; Dow, 2016; Karmali et al., 2021). These stereotypes may be particularly problematic when paired with ideas surrounding the superhumanization and dehumanization of Black men (Goff et al., 2008; Owusu-Bempah, 2017; Waytz et al., 2015). On the one hand, Black men are superhumanized, being perceived as more athletic, strong, and tough than other races (Madon et al., 2001; Wilson et al., 2017). At the same time, Black men are also dehumanized, being stereotyped as unintelligent and animalistic (Goff et al., 2008; Owusu-Bempah, 2017; Smith, 1990). Taken together, we argue that these stereotypes result in perceptions of Black men posing a threat of physical hostility. Specifically, animalistic and strength stereotypes are often associated with perceptions of Black men being involved in street-level crimes involving robberies and assault (Gordon et al., 1988; Quillian & Pager, 2001; Smiley & Fakunle, 2016).

## Arab Stereotypes

In the United States, Arabs have been repeatedly accused of being a threat to the American population. Despite a lack of evidence that Arab people were responsible for bombings in Oklahoma City in 1995 and Atlanta in 1996, and a plane crash in 1996, the media demonized this group of people (Kamalipour, 2000; Semaan, 2014). This negative image has persisted throughout entertainment media, where Arabs are often depicted as threats to American citizens (Kamalipour, 2000). Similar to Black people, Arab (and Muslim) men are considered the prototypical gender for their group and are more strongly associated with stereotypes related to threat than Arab (and Muslim) women (Erentzen et al., 2022; Saloom, 2006).

Similar to Black men, Arab men and Middle Eastern men are often stereotyped as aggressive, angry, cruel, hostile, uncivilized, unkind, and unsociable (Guterman, 2013, Karmali et al., 2021; Shaheen, 2003, Smith, 1990). However, in contrast to Black men, Middle Eastern men do not suffer from stereotypes of incompetence (Fiske et al., 2002; Sides & Gross, 2013; Timberlake & Williams, 2012). Further unlike Black men, Arab men are more likely to be perceived as carrying a threat of ideological, rather than physical, hostility. Specifically, Arab men are strongly associated with terrorism and religious fanaticism (Guterman, 2013; Saleem & Anderson, 2013; Saloom, 2006; Shaheen, 2003) rather than street-level crime. Therefore, compared with Black men, the hostile stereotype of Arab men likely stems from fears of terrorist attacks (i.e., an ideological threat) rather than being robbed or assaulted on the street (i.e., a physical threat).

## Intersectionality Research

Because people belong to multiple social groups, perceptions of a person may change when more than one of their identities is considered. There are different perspectives on how intersecting identities influence person perception. A double-jeopardy hypothesis claims that negative stereotypes across groups are additive (Barnum et al., 1995). This prediction fits with an error management approach, which suggests that people are likely to err on the side of over-perceiving threats, to minimize the potential costs of missing those threats (Haselton & Nettle, 2006; Nesse, 2005). Under this hypothesis, an older age or gay identity would not reduce any hostile stereotypes ascribed to Black or Arab men. However, research that has investigated the double-jeopardy hypothesis has found mixed evidence supporting it (Berdahl & Moore, 2006; Dowd & Bengston, 1978; Kang & Chasteen, 2009; Kang et al., 2014; Yap & Konrad, 2009).

A dominance hypothesis asserts that certain superordinate identities predominate person perception (Petsko & Bodenhausen, 2020). Prejudice is particularly directed toward individuals who fit the prototype of that category. For example, racial bias is directed toward young straight Black men, whereas gender bias is directed toward young straight White women. Resultantly, under this hypothesis, Black women, gay people, and older adults are considered chronically invisible and not the targets of prejudice (Petsko & Bodenhausen, 2020). Therefore, a dominance hypothesis would predict that older racial/ethnic minority men as well as gay racial/ethnic minority men would be stereotyped more positively than their younger or straight counterparts.

In contrast, intersectionality theory states that each person simultaneously belongs to multiple social categories that are mutually constitutive (Cole & Zucker, 2007). Therefore, stereotypes of different social groups and their downstream consequences are not additive. As a result, membership of multiple social categories gives rise to distinct stereotypes. To exemplify intersectionality theory, Ghavami and Peplau (2012) asked participants to report stereotypes most people hold about various superordinate racial groups stratified by gender (e.g., Black, Black men, and Black women). They found that many distinct stereotypes emerged for the subordinate gendered race categories compared with the superordinate race category. Under an intersectionality hypothesis, different intersectional identities may alter perceptions of threat compared with their superordinate male categories in different ways depending on the specific combinations of their identities (e.g., an older age identity may shift stereotype content differently for Arab men compared with Black men).

### Old Age Stereotypes

Stereotypes of older adults are nuanced, comprising a mix of positive and negative traits (Chasteen et al., 2018; Kite & Johnson, 1988). These traits include perceptions of older adults being warm, religious, generous, and old-fashioned, while also incompetent, feeble, dependent, sedentary, bitter, and more fixed in their traits than younger adults (Hummert et al., 1994; Neel & Lassetter, 2015). Within an aging context, older adult stereotypes have the potential to reduce the perception of perceived physical threat associated with stereotypes of Black people. For example, anger is perceived as appearing sooner and lasting longer in older White men compared with younger White men; however, the opposite pattern emerges for Black male targets (Kang & Chasteen, 2009; Kang et al., 2014). In this case, the angry stereotype for Black men is attenuated by an old age stereotype. This added complexity to person perception occurs when stereotypes of intersecting identities conflict (Kang et al., 2014). Specifically, the hostile stereotypes associated with Black men are offset by feeble and warmth stereotypes associated with older adults (Chasteen et al., 2002; Cuddy & Fiske, 2002).

As mentioned, although previous research has demonstrated that an old age identity reduces hostile stereotypes associated with Black men, there has not yet been an investigation of whether an old age identity reduces hostile stereotypes for other racial or ethnic groups. It is possible that if the hostile stereotype of Black men is driven by perceptions of physical threat that require a degree of athleticism, such as robberies and assault, then the feeble stereotype of older adults may be particularly well suited to counteract perceptions of Black men posing a threat. In contrast, if the hostile stereotype of Arab men is driven by perceptions of ideological threat, such as terrorism, then the feeble stereotype of older adults may not be well suited to counteract perceptions of Arab men posing a threat, because that form of threat does not necessarily require physical strength.

### Gay Stereotypes

In contrast to an older age identity, a gay identity may reduce hostile stereotypes for both male groups. As direct evidence for Black men, one study showed that while criminal, athletic, violent, aggressive, quick-tempered, and unintelligent were in the top 10 most representative traits for Black men, not a single one of these traits was considered highly representative of gay Black men (Petsko & Bodenhausen, 2019). Instead, stereotype content for gay men included traditionally feminine traits. Specifically, gay Black men, gay Asian men, and gay Hispanic men were stereotyped as sensitive, whereas gay White men, gay Asian men, and gay Hispanic men were stereotyped as delicate. As further indirect evidence, Remedios et al. (2011) reported that during an approach-avoidance task, participants instructed to approach Black men responded more quickly to gay Black men than straight Black men; the opposite pattern emerged for White men. These findings suggest that gay Black men may be stereotyped as less threatening as they age.

Based on past literature, a stronger case can be made that reductions in hostile stereotypes for gay Black men compared with straight Black men are a result of differences in perceptions of the psychological aspects of femininity (e.g., sensitivity) rather than physical strength. For example, no traits related to physical weakness were associated with gay Black men in Petsko and Bodenhausen (2019). Further, Madon (1997) demonstrated that gay men were stereotyped as good listeners, compassionate, touchy-feely, warm-hearted, feminine, affectionate, emotional, sensitive, soft hearted, and gentle. All these traits directly contrast hostile stereotypes associated with Black men, such as violent, aggressive, and quick-tempered. In that study, the only trait assigned to gay men that may be inferred as a sign of physical weakness was dainty (Madon, 1997). Taken together, findings from past literature suggest that a gay identity and its associated feminine stereotypes may neutralize the threat of physical hostility posed by Black men.

Evidence for whether a gay identity may attenuate perceptions of ideological hostility posed by Arab men is lacking. However, if people perceive conflict between being a religious fanatic and being gay, then we may expect that a gay identity may reduce both religious and hostile stereotypes that feed into perceptions of ideological hostility for Arab men. Considering that discrimination is driven by prejudice formed due to stereotypes (Plous, 2003), uncovering the presence of hostile stereotypes for underrepresented intersectional groups is an important step toward developing more nuanced discrimination reduction techniques.

## The Present Study

This study aimed to understand how the hostile stereotype of Black and Arab men changes when age and sexual orientation are taken into account. Because ‘Black’ includes people from a variety of geographic locations, we chose to use ‘Arab’ rather than ‘Middle Eastern’ to keep the group labels as similar as possible. We also collected stereotype content of White men; however, because our research question focuses specifically on comparisons between stereotypes about Arab and Black people, we omitted White stereotype data from this manuscript for conciseness (see Supplemental Material for a summary of results for White men data).

For our first research objective, we assessed whether the addition of an old age identity reduces hostile stereotype content more for Black men than Arab men. Given people’s inclinations to appear unprejudiced (Fazio et al., 1995), we used a common method of asking participants to report on societal stereotypes rather than their own beliefs (Bergstrom et al., 2022; Ghavami & Peplau, 2012; Petsko & Bodenhausen, 2019). Therefore, participants reported up to five stereotypes that they believed most people held about the following superordinate and intersecting identities: men, Arab men, Black men, old men, young men, old Arab men, young Arab men, old Black men, and young Black men.

Because Black men are perceived as physically threatening, an old age identity may attenuate negative stereotypes of hostility (Quillian & Pager, 2001). More specifically, perceptions of older adults being feeble, wise, and efficient at emotional regulation (Barrett & Cantwell, 2007; Lantrip & Huang, 2017; Robinson et al., 2009) may offset stereotypes of an older Black man being perceived as strong, unintelligent, and hot-tempered/aggressive, respectively. In contrast, because Arab men are associated with ideological threat, an old age identity might not successfully offset hostile stereotypes associated with Arab men. In fact, an old age identity may exacerbate stereotypes associated with terrorism and religious extremism if Americans associate these traits with middle-aged or older men like Osama bin Laden or Abdullah Azzam. Further, stereotypes related to older men being perceived as old-fashioned may further strengthen their association with ideological threat (Hummert et al., 1994). Therefore, we hypothesize that Arab men, compared with Black men, are less likely to “age out” of threatening stereotypes, because the type of hostility stereotypically associated with Arab men does not require physical strength.

For our second research objective, we assessed whether the addition of a gay identity reduces hostile stereotype content for both Black men and Arab men. We assessed perceptions of societal stereotypes for the following groups: men, Arab men, Black men, gay men, straight men, gay Arab men, straight Arab men, gay Black men, and straight Black men. We hypothesized that non-gender-conforming stereotypes associated with gay men, such as being sensitive and high in communion (Petsko & Bodenhausen, 2019; Steffens et al., 2019), may attenuate stereotypes of physical hostility (as posed by Black people); whereas a lack of religious extremism stereotypes associated with gay men may attenuate stereotypes related to ideological hostility (as posed by Arab people).

### Method

We obtained approval from the Ethics Research Board of the University of Toronto (Protocol #37031). To access the data and code for this project, visit https://doi.org/10.17605/OSF.IO/GM8X4.

### Participants

Based on prior research using the same study design (Bergstrom et al., 2022), we aimed to collect data from at least 80 participants per target group. Expecting exclusions, we oversampled: 600 Amazon Mechanical Turk (MTurk) participants were compensated US$1.00 for their participation in this study. The only inclusion criterion was that they must reside in the United States. We checked Internet Protocol (IP) addresses and geographic coordinates to ensure participants met this inclusion criteria. Duplicate IP addresses and worker IDs, participants who did not pass our attention item, and responses that were highly likely reported by bots (i.e., nonsensical multi-sentence responses) were excluded from analysis. The final sample size was 421 (82 to 89 participants provided stereotypes for each target group). The sample had a mean age of 35.32 (*SD* = 10.53) and 56.77% were male. The majority (76.96%) of participants were White, 9.03% were Black, 0.24% were Middle Eastern, and the remaining 13.78% identified with another racial group (see Supplemental Material Table S5 for full demographic breakdown).

### Procedure

Participants were asked to list up to five words or short phrases that most people hold about four of the following social groups: men, Arab men, Black men, old men, young men, old Arab men, young Arab men, old Black men, young Black men, gay men, straight men, gay Arab men, straight Arab men, gay Black men, and straight Black men (as well as White men categories, which we removed for conciseness). Groups were assigned to participants such that they never reported traits for any superordinate identity more than once. For example, 25% of participants were asked to report traits associated with young men, old Arab men, gay Black men, and straight White men. The presentation order of groups within each subset were randomized (see Supplemental Material for the five subsets of four groups that participants were assigned to). After reporting traits, participants responded to various demographic questions.

### Materials

#### Demographics

Participants reported their age, gender, political orientation, sexual orientation, race, religion, and highest level of education (see Supplemental Material to view participants’ full demographic breakdown).

### Data Analysis

Our coding process followed the procedure reported in Bergstrom et al. (2022). To start, two individual research assistants (RAs) cleaned the data. For phase 1, plural forms of words were changed to singular and typos were corrected. For phase 2, phrases were summarized into single words and then words were truncated to create meaningful roots that included all words that have a similar meaning (e.g., “terrorist” and “terrorism” to “terror*”). For phase 3, frequencies of each stereotype generated were recorded to create a stereotype dictionary for each superordinate and intersectional group. Disagreements through any phases were discussed between RAs. For cases of no resolution, the graduate supervisor was to be consulted; however, cases of no resolution did not occur.

Next, synonyms from the stereotype dictionaries were combined under a single term (e.g., “beautiful” became “attractive”). We used an online thesaurus (http://ww.thesaurus.com) to decide how to group words together. To make these changes, spreadsheets were loaded into Google’s Open Refine Program, which enabled all changes to be made across groups simultaneously in order to maintain consistency. Lastly, words that had a single count and were not listed for any other groups were dropped from analyses.

Statistical testing was conducted in R Studio (Version 1.3.959). Firstly, the percentage of overlap between the intersectional and superordinate categories was calculated based on all traits contained in the dictionaries for each group. Secondly, out of the top 10 stereotypes for each superordinate and intersectional group (i.e., Arab men, old Arab men, young Arab men, gay Arab men, straight Arab men, Black men, old Black men, young Black men, gay Black men, and straight Black men), we retained traits that were associated with hostility or that related to our hypotheses of physical or ideological threat for statistical testing. Specifically, this meant that in addition to hostile-related traits, we retained controlling, extreme, intolerant, Islamic, and religious because these traits may be relevant for perceptions of ideological threat (i.e., terrorism) posed by Arab men. Additionally, we retained athletic, degenerate, stupid, and tough because these traits may be relevant for perceptions of physical threat (i.e., street crime) posed by Black men. Fisher’s exact tests were performed on the retained traits to assess whether stereotype content shifted between the racial/ethnic groups and their old/young or gay/straight intersecting identities.

Due to overlap in stereotypes between old Arab/Black men, young Arab/Black men, and Arab/Black men as well as not every trait being associated with hostility or threat, the list of the top 10 stereotypes for each group (i.e., 30 stereotypes per racial group) was reduced to 12 tests for Arab men and 12 tests for Black men for the age group comparisons. Consequently, we Bonferroni-adjusted our alpha levels for the omnibus tests to .05/12 = .0042 for both groups. For sexual orientation comparisons, this resulted in 11 tests for Arab men and 9 tests for Black men. Therefore, we Bonferroni-adjusted our alpha levels for the omnibus tests to .05/11 = .0045 and .05/9 = .0056, respectively. When these omnibus tests were significant, we computed additional pairwise testing with a Holm adjustment to assess whether the stereotype content shift was the result of an old age identity, young age identity, or both for our first research objective or the result of a gay identity, straight identity, or both for our second research objective.

Lastly, to check that Black men were more likely to be stereotyped as physical threats than Arab men, we performed Fisher’s exact tests for athletic, degenerate, stupid, and tough. We Bonferroni-adjusted our alpha levels for the omnibus tests to .05/4 = .0125. Then, to test that Arab men were more likely to be stereotyped as ideological threats than Black men, we performed Fisher’s exact tests for controlling, extreme, intolerant, Islamic, and religious. We Bonferroni-adjusted our alpha levels for the omnibus tests to .05/5 = .0100.

### Results

#### Stereotype Content Overlap

##### Age

With the superordinate men category, young men had the greatest amount of overlap, followed by Black men, Arab men, and old men (see Table 1 for specific percentage overlap). As expected, young Black men, compared with old Black men, had more stereotype content overlap with Black men. In contrast, young Arab men, compared with old Arab men, had less stereotype content overlap with Arab men (although the difference was relatively small at 2.70%; see Table 2 for specific percentage overlap).

**Table 1.** Stereotype content overlap with the men category.

Subgroup	Superordinate group	Overlap (%)
Arab men	Men	40.00
Black men	Men	42.35
Old men	Men	34.12
Young men	Men	63.53
Gay men	Men	44.71
Straight men	Men	70.59

**Table 2.** Stereotype content overlap with the Arab or Black men category.

Subgroup	Superordinate group	Overlap (%)
Old Arab men	Arab men	71.62
Young Arab men	Arab men	68.92
Old Black men	Black men	60.92
Young Black men	Black men	68.97
Gay Arab men	Arab men	61.84
Straight Arab men	Arab men	77.63
Gay Black men	Black men	66.67
Straight Black men	Black men	63.21

##### Sexual orientation

With the superordinate men category, straight men had the greatest amount of overlap, followed by gay men, Black men, and Arab men (see Table 1 for specific percentage overlap). As expected, straight Arab men, compared with gay Arab men, had more stereotype content overlap with Arab men. Unexpectedly, gay Black men, compared with straight Black men, had more stereotype content overlap with Black men (although the difference was relatively small at 3.46%; see Table 2 for specific percentage overlap).

#### Fisher’s exact tests

##### Physical threat

Among the four tested traits, Black men were stereotyped as more athletic, stupid, and tough than Arab men. The test for degenerate had a *p*-value of .014, which failed to reach our Bonferroni-adjusted alpha level of .0125 (see Table 3 for Fisher’s exact tests).

**Table 3.** Fisher’s exact results for physical threat stereotype content comparisons.

Word	Arab men	Black men	Fisher’s exact test *p*-value
Athletic	0	23	< .001
degenerate	1	7	.014
stupid	2	25	< .001
tough	1	13	.001

*Note.* The Bonferroni-adjusted alpha level was .0125; this table includes frequency counts of generated traits across all participants.

##### Ideological threat

Among the five tested traits, Arab men were stereotyped as more controlling, extreme, intolerant, Islamic, and religious than Black men (see Table 4 for Fisher’s exact tests).

**Table 4.** Fisher’s exact results for ideological threat stereotype content comparisons.

Word	Arab men	Black men	Fisher’s exact test *p*-value
controlling	16	2	< .001
extreme	11	0	< .001
intolerant	34	1	< .001
Islamic	21	0	< .001
religious	22	1	< .001

*Note.* The Bonferroni-adjusted alpha level was .0100; this table includes frequency counts of generated traits across all participants.

##### Age

We report on the top five stereotypes for each group. When there was a tie for fifth place, we report all traits included in the tie.

##### Arab men

The top five stereotypes for Arab men were intolerant, terrorist, religious, Islamic, and dangerous; the top five stereotypes for old Arab men were intolerant, terrorist, conservative, religious, Islamic, and filthy; the top five stereotypes for young Arab men were terrorist, filthy, intolerant, Islamic, and religious. Among the 12 traits tested, an age intersecting identity shifted stereotype content for only 2 traits: terrorist and controlling. Specifically, a young age identity decreased perceptions of being controlling while increased perceptions of terrorism. An old age identity did not shift stereotype content away from the superordinate Arab man category for any of the traits (see Table 5 for Fisher’s exact tests).

**Table 5.** Fisher’s exact results for Arab stereotype content age comparisons.

Word	Arab men	Old Arab men	Young Arab men	Fisher’s exact test *p*-value
angry	4	5	9	.392
controlling	16_a_	7_a,b_	1_b_	< .001
dangerous	18	8	13	.109
extreme	11	3	11	.049
intolerant	34	39	23	.027
Islamic	21	13	23	.154
mean	9	8	8	.965
religious	22	16	19	.541
stupid	2	9	9	.063
terrorist	33_a,b_	23_a_	45_b_	.003
untrustworthy	7	8	12	.523
violent	14	8	13	.357

*Note.* Groups that share subscripts indicate a nonsignificant difference; this table includes frequency counts of generated traits across all participants.

##### Black men

The top five stereotypes for Black men were stupid, lazy, athletic, threatening, violent, and immoral; the top five stereotypes for old Black men were intelligent, cranky, poor, intolerant, and lazy; the top five stereotypes for young Black men were criminal, stupid, immoral, athletic, and lazy. Among the 12 tested traits, an old age intersecting identity shifted stereotype content for 9 traits, increasing perceptions of intolerance and decreasing perceptions of athleticism, criminality, dangerousness, immorality, stupidity, threat, toughness, and violence. In contrast, a young age identity shifted stereotype content away from the superordinate Black man category for only one trait: increasing perceptions of criminality (see Table 6 for Fisher’s exact tests).

**Table 6.** Fisher’s exact results for Black stereotype content age comparisons.

Word	Black men	Old Black men	Young Black men	Fisher’s exact test *p*-value
angry	10	10	6	0.593
Athletic	23_a_	1_b_	23_a_	< .001
criminal	19_a_	4_b_	45_c_	< .001
dangerous	11_a_	2_b_	14_a_	.003
degenerate	7	9	14	.174
immoral	20_a_	0_b_	27_a_	< .001
intolerant	1_a_	16_b_	0_a_	< .001
mean	4	9	5	.297
stupid	25_a_	10_b_	35_a_	< .001
threat	21_a_	3_b_	13_a_	< .001
tough	13_a_	1_b_	7_a,b_	.003
violent	20_a_	0_b_	13_a_	< .001

*Note.* Groups that share subscripts indicate a nonsignificant difference; this table includes frequency counts of generated traits across all participants.

##### Sexual orientation

We report on the top five stereotypes for each group. When there was a tie for fifth place, we report all traits included in the tie.

##### Arab men

The top five stereotypes for Arab men were intolerant, terrorist, religious, Islamic, and dangerous; the top five stereotypes for straight Arab men were terrorist, intolerant, violent, Islamic, and religious; the top five stereotypes for gay Arab men were closeted, terrorist, ostracized, dangerous, intolerant, and filthy. Among the 11 tested traits, a gay identity shifted stereotype content for 8 traits: decreasing perceptions of being controlling, extreme, intolerant, Islamic, mean, religious, a terrorist, and violent compared with Arab men. However, it did not alter perceptions of dangerousness, rigidness, or untrustworthiness. A straight identity did not shift stereotype content away from the superordinateArab man category for any of the traits (see Table 7 for Fisher’s exact tests).

**Table 7.** Fisher’s exact results for Arab stereotype content sexual orientation comparisons.

Word	Arab men	Gay Arab men	Straight Arab men	Fisher’s exact test p-value
controlling	16_a_	1_b_	4_a_	< .001
dangerous	18	11	15	.370
extreme	11_a_	0_b_	15_a_	< .001
intolerant	34_a_	10_b_	28_a_	< .001
Islamic	21_a_	3_b_	19_a_	< .001
mean	9_a_	2_b_	8_a_	< .001
religious	22_a_	4_b_	18_a_	< .001
rigid	4	0	9	.006
terrorist	33_a_	15_b_	35_a_	.003
untrustworthy	7	2	11	.041
violent	14_a_	1_b_	22_a_	< .001

*Note.* Groups that share subscripts indicate a nonsignificant difference; this table includes frequency counts of generated traits across all participants.

##### Black men

The top five stereotypes for Black men were stupid, lazy, athletic, threat(ening), violent, and immoral; the top five stereotypes for straight Black men were athletic, stupid, immoral, threatening, criminal, tough, and lazy; the top five stereotypes for gay Black men were feminine, fashionable, excessive, filthy, and loud. Among the nine tested traits, a gay identity shifted stereotype content for six traits, decreasing perceptions of being athletic, criminal, immoral, stupid, threatening, and violent compared with Black men. However, it did not shift stereotype content away from the superordinate Black men category for intolerant, rude, or tough, but, notably, intolerant was a seldomly reported trait for both groups. A straight identity shifted stereotype content for a single trait, increasing perceptions of being intolerant (see Table 8 for Fisher’s exact tests).

**Table 8.** Fisher’s exact results for Black stereotype content sexual orientation comparisons.

Word	Black men	Gay Black men	Straight Black men	Fisher’s exact test *p*-value
athletic	23_a_	2_b_	20_a_	< .001
criminal	19_a_	0_b_	15_a_	< .001
immoral	20_a_	3_b_	18_a_	< .001
intolerant	1_a_	2_a_	12_b_	< .001
rude	6	7	3	.447
stupid	25_a_	5_b_	19_a_	< .001
threat	21_a_	2_b_	16_a_	< .001
tough	13	4	15	.020
violent	20_a_	0_b_	13_a_	< .001

*Note.* Groups that share subscripts indicate a nonsignificant difference; this table includes frequency counts of generated traits across all participants.

## Discussion

The present study assessed how intersectional identities shift stereotype content for Black and Arab men, particularly with respect to stereotypes related to hostility.

### How Age Affects Stereotype Content

Due to the hostile stereotype of Black men being associated with physical threat (Gordon et al., 1988; Quillian & Pager, 2001), we hypothesized that old age stereotypes such as being frail, wise, and efficient at emotional regulation (Barrett & Cantwell, 2007; Lantrip & Huang, 2017; Robinson et al., 2009) would reduce the hostile stereotype for Black men. In contrast, due to the hostile stereotype of Arab men being associated with ideological threat (Guterman, 2013; Saleem & Anderson, 2013; Shaheen, 2003), we hypothesized that old age stereotypes would not reduce the hostile stereotype for Arab men.

To test this hypothesis, we qualitatively identified stereotype content for Arab men, old Arab men, young Arab men, Black men, old Black men, and young Arab men. Our data supported the assertion that the hostility stereotype for Black men and Arab men differ. As expected, an old age identity offset hostile stereotypes for Black men, but not Arab men. In particular, results showed that an old age identity reduced stereotypes of Black men being perceived as athletic, criminal, dangerous, immoral, stupid, threatening, tough, and violent. In contrast, an old age identity did not reduce stereotypes of Arab men being perceived as angry, controlling, dangerous, mean, stupid, a terrorist, untrustworthy, violent, extreme, intolerant, Islamic, or religious. However, it should be highlighted that a young age exacerbated perceptions of terrorism without increasing perceptions of any other hostility-related traits. We did not anticipate this one result, making it worthy of future investigation.

In further support of our hypothesis, young Black men, compared with old Black men, had descriptively more stereotype content overlap with Black men (68.97% vs. 60.92%, respectively). These results suggest that when people think about the superordinate Black men category, they are more likely to think about young Black men compared with old Black men. Therefore, the stereotypes that previous research has uncovered related to Black men are more likely to apply to young Black men than old Black men.

In contrast, young Arab men, compared with old Arab men, had descriptively less stereotype content overlap with Arab men (68.92% vs. 71.62%, respectively). These results suggest that when people think about the superordinate Arab men category, they are slightly more likely to think about old Arab men than young Arab men. Taken together, our finding that there was a greater difference in overlap with the superordinate racial group for young and old Black than for young and old Arab men suggests that an old age identity is more likely to shift stereotype content for Black men compared with Arab men. However, it is important to keep in mind that these results are descriptive and need to be replicated.

In sum, an old age identity appears to offset stereotypes associated with physical threat for Black men, but not stereotypes associated with ideological threat for Arab men. Therefore, our findings show that compared to Arab men, Black men are less likely to be stereotyped as threatening as they age.

### How Sexual Orientation Affects Stereotype Content

Unlike our divergent predictions for how old age would influence hostility stereotypes for the two male groups, we expected that a gay identity would offset hostile stereotypes regardless of whether these stereotypes were driven by feelings of physical threat (as posed by Black men) or ideological threat (as posed by Arab men). This is because gay stereotypes are at odds with both physical street-level crime, national- or international-level crime, and religious fanaticism.

To test this hypothesis, we qualitatively identified stereotype content for Arab men, gay Arab men, straight Arab men, Black men, gay Black men, and straight Black men. In this case, a gay identity offset hostile stereotypes for both male groups. In particular, a gay identity reduced stereotypes of Black men being perceived as athletic, criminal, immoral, stupid, threatening, and violent; and reduced stereotypes of Arab men being perceived as controlling, extreme, intolerant, Islamic, mean, religious, a terrorist, and violent. In general, a gay identity appears to offset stereotypes associated with street-level threat for Black men as well as stereotypes associated with terrorist-level threat for Arab men, with a few exceptions (i.e., perceptions of being rude and tough remained unaltered for Black men, while perceptions of dangerousness, rigidness, and untrustworthiness remained unaltered for Arab men).

Past literature has provided more support for any reduction in hostile stereotypes for gay Black men compared with straight Black men being a result of differences in perceptions of the psychological aspects of femininity (e.g., sensitivity) rather than physical strength (Madon, 1997; Petsko and Bodenhausen, 2019). In the present study, only two traits associated with physicality were generated by participants: athletic and tough. A gay identity reduced stereotypes of athleticism, but not toughness. Therefore, we found mixed evidence as to whether a gay identity may reduce perceptions of physical strength for Black men. More research is required to further explore this idea. Overall, our findings show that compared with their superordinate male category, gay Black and gay Arab men are more likely to be stereotyped as less threatening.

In line with our hypothesis, straight Arab men, compared with gay Arab men, had descriptively more stereotype content overlap with Arab men (77.63% vs. 61.84%, respectively). These results suggest that when people think about the superordinate Arab men category, they are more likely to think about straight Arab men compared with gay Arab men. Therefore, the stereotypes that previous research has uncovered related to Arab men are more likely to apply to straight Arab men than gay Arab men.

Unexpectedly, straight Black men, compared with gay Black men, had descriptively less stereotype content overlap with Black men (63.21% vs. 66.67%, respectively). These findings are at odds with dominance models, which predict that straight men are most prototypical of the Black men category (Petsko & Bodenhausen, 2019). Future research will need to assess whether gay Black men share more stereotypes in common with Black men than straight Black men as a result of increased prototypicality for their superordinate group or for some other reason.

Regarding the method we used to assess stereotype content overlap, we included *all* traits that at least two participants generated for one of the target groups. In other words, the stereotype content overlap data included more than the top 10 to 20 traits for each group. It is likely that stereotype content for the *most common* traits assigned to straight Black men and the superordinate Black men category overlap more than between gay Black men and the superordinate category. For example, straight Black men and Black men shared eight out of the top nine traits related to physical hostility, whereas gay Black men and straight Black men only shared two of these same traits. However, gay Black men and Black men shared some traits that were rarely reported for the superordinate category and never reported for straight Black men, such as carefree, entitled, fashionable, and powerless. Thus, these less frequently reported traits might have led to a greater overlap between the category gay Black men and the superordinate Black men category, and also the content of these less frequent traits (e.g., carefree, powerless) may have offset hostile stereotypes associated with Black men.

In sum, a gay identity appears to offset stereotypes associated with physical threat for Black men, as well as stereotypes associated with ideological threat for Arab men. Therefore, our findings show that gay Arab men and gay Black men are less likely to be stereotyped as threatening than their straight counterparts.

### Theoretical Implications

Overall, our findings provided more support for an intersectionality hypothesis (Cole & Zucker, 2007) rather than a double-jeopardy hypothesis (Haselton & Nettle, 2006) or dominance hypothesis (Petsko & Bodenhausen, 2020). Under a double-jeopardy hypothesis, gay Black men, old Black men, gay Arab men, and old Arab men would be stereotyped as threatening as their younger and straight counterparts. Our findings did not support this hypothesis as old Black men, gay Black men, and gay Arab men were stereotyped as less threatening than their superordinate male category. Under a dominance hypothesis, older male minority group members as well as gay minority group members would be stereotyped more positively compared with their younger or straight counterparts. Our findings did not support this hypothesis as old Arab men were stereotyped to be as threatening as their superordinate male category. Finally, under an intersectionality hypothesis, different intersectional identities may alter stereotypes related to threat compared with their superordinate men categories in different ways. Our findings supported this hypothesis as an old age identity, compared with a gay identity, did not offset hostility-related stereotypes for Arab men as it did for Black men.

The present studies suggest that different considerations will thus need to be taken into account when creating interventions to reduce discrimination. For example, interventions to reduce an irrational fear of Black men may be more effective when focused on improving stereotypes of young Black men compared with old Black men; however, interventions to reduce an irrational fear of Arab men may need to include all male age groups to be the most effective.

### Limitations

This study was a novel exploration into how hostile stereotypes shift for Black and Arab men when age or sexual orientation is considered. However, there were some limitations. Firstly, we asked participants to report stereotypes held by most people instead of their personal beliefs to overcome social desirability bias. Therefore, these findings may represent knowledge of existing stereotypes rather than personal endorsement. Secondly, the lack of diversity in our participant pool (~75% White) limited our ability to investigate the impact of participant race on stereotype content. Results may differ based on participant race or ethnicity due to in-group favoritism leading participants to rate their in-groups more favorably, potentially generating more positive stereotype content for Black and Arab people.

Due to the qualitative nature of data collection for this research and the coding method that resulted in a table of stereotyped frequencies as the data structure, group membership of the participants could not be retained for covariate purposes either. However, because over 90% of our sample was non-Black and non-Arab and over 95% of our sample did not identify as gay, retaining these people in the sample may add some noise, but it is unlikely that any of the conclusions we have drawn would change. Because our research question relates to generalized society-wide stereotypes in the United States and Black, Arab, and gay people are members of American society, we believed that it was important to retain them in our sample.

Thirdly, groups were assigned to participants such that they never reported traits for any superordinate identity more than once. For example, 25% of participants were asked to report traits associated with young men, old Arab men, gay Black men, and straight White men. We believed that if the same participant were to report stereotypes for two groups that shared membership for a particular identity (e.g., old Arab men and old Black men share being old; young Arab men and old Arab men share being Arab), their responses would be influenced more by the preceding group than if they were to report stereotypes for groups comprising different memberships (e.g., old Arab men and gay Black men do not share membership on sexual orientation, age, or race). Thus, our rationale for providing participants with groups that had non-overlapping racial, age, and sexual orientation identities represented our efforts to reduce any potential priming effects of responding to multiple identities.

However, not fully randomizing the design means that the particular sets of groups that participants saw were held constant across the sample. For example, every participant who responded to old Arab men also responded to gay Black men. Viewing one of these groups always with another group may have led to a priming for, or contrast away from, these categories. In order to mitigate the repercussions of this possibility, we not only randomized which set of groups that participants saw, but also randomized the order of groups within each subset.

Lastly, we used https://www.thesaurus.com to help combine synonyms and reduce our trait list. For example, biased, prejudiced, and racist were recategorized to intolerant because these traits are strongly associated with one another based on thesaurus.com. However, some traits went through more complex paths to be recategorized. For example, misogynist was moderately associated with sexist on thesaurus.com, and then sexist was strongly associated with intolerant. Further, there were times where participants reported phrases to describe a group, such as “kills anyone who disagrees with them,” that were not able to be entered into thesaurus.com. For such cases, the research team had to use their judgment to decide how to recategorize the trait.

Additionally, we made some executive decisions on when to keep certain traits separate. For example, intolerant is strongly associated with fanatical and fanatical is moderately associated with extreme on thesaurus.com. We chose to recategorize fanatical as extreme and keep this trait separate from intolerant. We acknowledge that other researchers may have made different decisions but that is the nature of collecting qualitative data. To ensure that our own biases were not affecting how synonyms were grouped together, our spreadsheets for each target group were loaded into Google’s Open Refine Program, which enabled all changes to be made across groups simultaneously, maintaining consistency.

### Future Directions

The present research focused on intersecting identities of men. Future research will need to examine how stereotypes shift when age or sexual orientation are taken into account for Arab and Black women. We do not expect that Arab and Black women are stereotyped as hostile to the same degree as men. As loosely related evidence, we found in our recent research that top stereotypes for Muslim men include terrorist, strict, radical, violent, and aggressive (Erentzen et al., 2022). The only one of these top stereotypes that Muslim women shared with Muslim men was terrorist. In contrast, Muslim women were stereotyped as oppressed, submissive, quiet, and meek. If Arab women are stereotyped similarly to Muslim women, then we would not expect our hypotheses for the present study to hold for Arab women because there is not a hostile stereotype for an older age or gay identity to offset.

Further, we collected perceptions of baseline stereotypes, meaning that participants were not primed to think about the target groups within a specific context. We did this so that future researchers may look at our complete list of traits (see https://doi.org/10.17605/OSF.IO/GM8X4) for all groups to help generate new hypotheses. However, stereotypes of intersectional groups may vary across time because the lenses that people use to perceive outgroup members can be intersectional (e.g., a race-by-gender lens) in certain situations, whereas singular (e.g., gender-only lens) in other situations (Petsko et al., 2022). Therefore, future studies will need to explore how hostile stereotypes of Arab and Black men change across contexts or perceiver goals.

Future studies should aim to understand how stereotypes of these groups differ based on participant race and whether particular minority groups endorse the same stereotype content toward outgroup minority groups as majority group members. Additionally, future research should assess how stereotype content shifts when people are asked to reflect on the traits held by Arabs, Middle Easterners, or Muslims. Lastly, it is important to keep in mind that we measured stereotypes in the present study. Future studies are needed to assess the implications of these stereotypes for evaluations of Black and Arab men.

## Conclusion

Because individuals belong to many social groups simultaneously, it is important to develop a more nuanced understanding of how different identities interact to influence stereotype content. With a better understanding of the negative stereotypes that intersectional groups face, we can create more targeted interventions aimed at reducing the pervasive discrimination that Black and Arab people face in North America. This paper is one of the few that assesses intersectionality by race, age, and sexual orientation and its findings are important because they suggest that Americans perceive intersectionality enough to alter their stereotypes of groups. Therefore, this work provides a layer of nuance to the prejudice and stereotyping literature with respect to homogeneity being a precursor of bias. As such, it represents an important first step in uncovering how stereotype content changes when additional identities are used as added lenses to inform person perception.

## Supplemental Material

sj-docx-1-gpi-10.1177_13684302231153802 – Supplemental material for Same view, different lens: How intersectional identities reduce Americans’ stereotypes of threat regarding Arab and Black men Click here for additional data file. Supplemental material, sj-docx-1-gpi-10.1177_13684302231153802 for Same view, different lens: How intersectional identities reduce Americans’ stereotypes of threat regarding Arab and Black men by Veronica N. Z. Bergstrom, Jonathan Cadieux, Drishti Thakkar and Alison L. Chasteen in Group Processes & Intergroup Relations

## Notes

1. In our introduction we present research on perceptions of both Arabs and Middle Easterners. As described by TeachMideast (An Educational Initiative of the Middle East Policy Council), Arabs are people whose first language is Arabic. There are 26 countries (e.g., Saudi Arabi, United Arab Emirates, Egypt) that have Arabic as one of their official languages. In contrast, Middle Easterners are people who come from a geographic area of the globe (approximately 26 countries). Depending on the characteristics used to define the Middle East, its border is dynamic. Although Arab and Middle Eastern countries have a high degree of overlap, there can be some discrepancies. For example, Turkey may be described as a non-Arab Middle Eastern country, whereas Morocco may be described as an Arab country outside the Middle East. More research is needed to determine whether any divergent stereotype content emerges depending on whether participants are asked to evaluate Arabs or Middle Easterners. Further, research is needed to determine whether stereotype content changes depending on the characteristics used to define the Middle East. For more information on the differences between Arabs, Middle Easterners, and Muslims, visit https://teachmideast.org/articles/arab-middle-eastern-and-muslim-whats-the-difference/

2. All literature cited in our introduction comes from the United States, except Kang and Chasteen (2009) and Remedios et al. (2011), where data collection occurred in Canada, as well as Steffens et al. (2019), where data collection occurred in Germany. It is important to keep in mind that our hypotheses are likely only applicable to western countries, where White people have a long history of being the majority group in the nation.

3. We recognize that Muslims also share these same stereotypes with Arabs. However, in this paper we focus solely on research that has investigated stereotypes of Arabs or Middle Easterners in order to compare how intersecting identities influence perceptions of two racial/ethnic groups rather than a racial group and a religious group.
