# Supplementary material for: Same view, different lens: How intersectional identities reduce Americans’ stereotypes of threat regarding Arab and Black men
Source: Group Process Intergroup Relat. 2023 Feb 22;27(2):348–65. doi: 10.1177/13684302231153802 (PMC10857900; doi:10.1177/13684302231153802)
Supplement: sj-docx-1-gpi-10.1177_13684302231153802 – Supplemental material for Same view, different lens: How intersectional identities reduce Americans’ stereotypes of threat regarding Arab and Black men [file sj-docx-1-gpi-10.1177_13684302231153802.docx]

**Supplemental Material**

**Same View, Different Lens: How Intersectional Identities Reduce Perceptions of Threat Toward Arab and Black Men**

This is the supplemental material for “Same view, different lens: How intersectional identities reduce perceptions of threat toward Arab and Black men.” This document contains demographic information about the sample, details on how participants were assigned to condition, and additional results for the White men category.

Table of Contents

[Results for White Men 2](#_Toc120780567)

[Participant Demographics 5](#_Toc120780568)

[Participant Conditions 6](#_Toc120780569)

# Results for White Men

**Stereotype Content Overlap**

Table S1.

*Study 1a Stereotype Content Overlap with the Men Category*

| **Subgroup** | **Superordinate group** | **Overlap (%)** |
| --- | --- | --- |
| Arab men | Men | 40.00 |
| Black men | Men | 42.35 |
| White men | Men | 62.35 |
| Old Men | Men | 34.12 |
| Young Men | Men | 63.53 |
| Gay Men | Men | 44.71 |
| Straight Men | Men | 70.59 |

Table S2.

*Study 1a Stereotype Content Overlap with the White Men Category*

| **Subgroup** | **Superordinate group** | **Overlap (%)** |
| --- | --- | --- |
| Old White men | White men | 41.58 |
| Young White men | White men | 70.30 |
| Gay White men | White men | 36.63 |
| Straight White men | White men | 65.35 |

**Age Results for White men**

The top five stereotypes for White men were intolerant, privileged, financially secure, intelligent, and arrogant; the top five stereotypes for old White men were intolerant, cranky, conservative, financially secure, and selfish; the top five stereotypes for young White men were intolerant, intelligent, privileged, entitled, and kind. Because White men are not associated with hostility, we tested the top ten traits for each group rather than traits associated with hostility. Among the 19 traits tested, an old age identity shifted stereotype content for six traits, increasing perceptions of conservatism, crankiness, forgetfulness, frailty, slowness, and decreasing perceptions of being privileged. A young age stereotype also decreased perceptions of being privileged, but did not shift any other stereotypes relative to the superordinate White men category. (see Table S3 for Fisher’s Exact tests).

Table S3.

*Fisher’s Exact Results for White Stereotype Content Age Comparisons*

| Word | White men | Old White men | Young White men | Fisher’s exact test p-value |
| --- | --- | --- | --- | --- |
| angry | 3 | 9 | 2 | .042 |
| conservative | 4_a_ | 24_b_ | 6_a_ | < .001 |
| cranky | 0_a_ | 25_b_ | 0_a_ | < .001 |
| entitled | 7 | 4 | 14 | .059 |
| financially secure | 25 | 19 | 10 | .007 |
| forgetful | 0_a_ | 10_b_ | 0_a_ | < .001 |
| frail | 0_a_ | 10_b_ | 0_a_ | < .001 |
| hardworking | 9 | 0 | 8 | .003 |
| intelligent | 20 | 7 | 17 | .017 |
| intolerant | 39_a,b_ | 54_a_ | 29_b_ | < .001 |
| kind | 9 | 10 | 12 | .889 |
| lazy | 2 | 6 | 10 | .074 |
| mean | 1 | 9 | 4 | .023 |
| privileged | 29_a_ | 4_b_ | 16_c_ | < .001 |
| rude | 4 | 4 | 10 | .187 |
| selfish | 10 | 12 | 9 | .701 |
| slow | 0_a_ | 11_b_ | 1_a_ | < .001 |
| stupid | 2 | 8 | 11 | .041 |
| successful | 9 | 1 | 5 | .025 |

*Note.* Groups that share subscripts indicate a nonsignificant difference.

**Sexual Orientation Results for White men**

The top five stereotypes for White men were intolerant, privileged, financially secure, intelligent, and arrogant; the top five stereotypes for gay White men were feminine, excessive, fashionable, loud, and kind; the top five stereotypes for straight White men were intolerant, privileged, arrogant, intelligent, boring, and conservative. Because White men are not associated with hostility, we tested the top ten traits for each group rather than traits associated with hostility. Among the 27 traits tested, a gay identity shifted stereotype content for 12 traits, increasing perceptions of excessiveness, being fashionable, femininity, homosexuality, loudness, and promiscuity, while decreasing perceptions of arrogance, being financially secure, intelligence, intolerance, being privileged, and selfishness. A straight identity shifted stereotype content by reducing perceptions of being financially secure (see Table S4 for Fisher’s Exact tests).

Table S4.

*Fisher’s Exact Results for White Stereotype Content Sexual Orientation Comparisons*

| Word | White men | Gay White men | Straight White men | Fisher’s exact test p-value |
| --- | --- | --- | --- | --- |
| arrogant | 20_a_ | 0_b_ | 17_a_ | < .001 |
| athletic | 0 | 1 | 7 | .004 |
| average | 3 | 0 | 7 | .014 |
| boring | 5_a,b_ | 0_a_ | 11_b_ | < .001 |
| conservative | 4 _a,b_ | 0_a_ | 11_b_ | < .001 |
| controlling | 4 | 0 | 8 | .005 |
| emotional | 1 | 7 | 0 | .006 |
| entitled | 7 | 0 | 4 | .010 |
| excessive | 0_a_ | 33_b_ | 0_a_ | < .001 |
| fashionable | 2_a_ | 26_b_ | 0_a_ | < .001 |
| feminine | 0_a_ | 47_b_ | 0_a_ | < .001 |
| financially secure | 25_a_ | 4_b_ | 10_b_ | < .001 |
| hardworking | 9 | 0 | 6 | .002 |
| homosexual | 0_a_ | 8_b_ | 0_a_ | < .001 |
| intelligent | 20_a_ | 1_b_ | 13_a_ | < .001 |
| intolerant | 39_a_ | 4_b_ | 49_a_ | < .001 |
| kind | 9 | 12 | 5 | .213 |
| loud | 1_a_ | 13_b_ | 1_a_ | < .001 |
| masculine | 3 | 0 | 7 | .014 |
| powerful | 6 | 0 | 7 | .012 |
| privileged | 29_a_ | 3_b_ | 33_a_ | < .001 |
| promiscuous | 0_a_ | 9_b_ | 1_a_ | < .001 |
| rude | 4 | 7 | 6 | .738 |
| selfish | 10_a_ | 0_b_ | 8_a_ | .001 |
| successful | 9 | 0 | 7 | .003 |
| tough | 6 | 1 | 7 | .079 |
| weak | 4 | 7 | 1 | .096 |

*Note.* Groups that share subscripts indicate a nonsignificant difference.

# Participant Demographics

Table S5.

| Characteristic | Percentage |
| --- | --- |
| **Gender** |  |
| Male | 56.77 |
| Female | 42.99 |
| Prefer not to say | 0.24 |
| **Race** |  |
| White | 76.96 |
| Black | 9.03 |
| Latin, Central, and South American | 4.99 |
| East/Southeast Asian | 3.56 |
| Multiracial | 3.56 |
| South Asian | 1.19 |
| Aboriginal/Indigenous | 0.24 |
| Middle Eastern | 0.24 |
| Other | 0.24 |
| **Religion** |  |
| Christian | 42.28 |
| Agnostic | 22.33 |
| Atheist | 18.05 |
| None | 11.16 |
| Buddhist | 2.38 |
| Views from multiple religions | 1.19 |
| Islamic | 0.47 |
| Hindu | 0.24 |
| Jewish | 0.24 |
| Other | 1.66 |
| **Sexual Orientation** |  |
| Straight | 86.94 |
| Bisexual | 8.08 |
| Gay | 3.80 |
| Other | 0.71 |
| Prefer not to say | 0.48 |
| **Education** |  |
| Did not complete High School | 0.24 |
| High school graduate/GED | 35.86 |
| Bachelor’s degree, college diploma, or associate’s degree | 54.87 |
| Masters, Doctor, or Professional degree | 9.02 |
| **Political Orientation** |  |
| Liberal | 49.88 |
| Conservative | 29.93 |
| Neutral | 20.19 |
| **ESL** |  |
| English as first language | 98.81 |
| English as second language | 1.19 |

# Participant Conditions

Participants were randomly assigned to report traits for one of the following subsets of the 20 groups.

1. Young men, old Arab men, gay Black men, straight White men
2. Straight men, old Black men, young Arab men, gay White men
3. Men, straight Black men, old White men, Arab men
4. White men, gay Arab men, young Black men, old men
5. Young White men, Black men, straight Arab men, gay men

The order of the groups within each subset was randomized.
